# Synthesis and Biological Evaluation of 2,4-Diaminopyrimidine-Based Antifolate Drugs against *Bacillus anthracis*

**DOI:** 10.3390/molecules19033231

**Published:** 2014-03-17

**Authors:** Baskar Nammalwar, N. Prasad Muddala, Christina R. Bourne, Mary Henry, Philip C. Bourne, Richard A. Bunce, Esther W. Barrow, K. Darrell Berlin, William W. Barrow

**Affiliations:** 1 Department of Chemistry, Oklahoma State University, 107 Physical Sciences, Stillwater, OK 74078, USA; E-Mails: baskar@okstate.edu (B.N.); muddala@okstate.edu (N.P.M.); kdb@okstate.edu (K.D.B.); 2 Department of Veterinary Pathobiology, Oklahoma State University, 250 McElroy Hall, Stillwater, OK 74078, USA; E-Mails: cbourne@ou.edu (C.R.B.); henrym@okstate.edu (M.H.); pcbourne@yahoo.com (P.C.B.); esther.barrow@okstate.edu (E.W.B.); bill.barrow@okstate.edu (W.W.B.)

**Keywords:** Gram-positive bacteria, *Bacillus anthracis*, 2,4-diaminopyrimidine, Heck reaction, antibiotic resistance, dihydrofolate reductase (DHFR), antifolates

## Abstract

Due to the innate ability of bacteria to develop resistance to available antibiotics, there is a critical need to develop new agents to treat more resilient strains. As a continuation of our research in this area, we have synthesized a series of racemic 2,4-diaminopyrimidine-based drug candidates, and evaluated them against *Bacillus anthracis.* The structures are comprised of a 2,4-diaminopyrimidine ring, a 3,4-dimethoxybenzyl ring, and an *N*-acryloyl-substituted 1,2-dihydrophthalazine ring. Various changes were made at the C1 stereocenter of the dihydrophthalazine moiety in the structure, and the biological activity was assessed by measurement of the MIC and K_i_ values to identify the most potent drug candidate.

## 1. Introduction

The growing problem of antibiotic resistance is prominent in medical reports and the scientific literature, which highlight the emergence of multidrug resistant bacteria [1,2]. For example, *Bacillus anthracis* continues to be one of the most fatal pathogens to humans and has become a major concern due to its potential use as a bioterrorism weapon [3]. The threat of bioterrorism arises from dormant spores of *B. anthracis*, which can readily germinate into an infectious form upon inhalation [4]. Like other Gram-positive bacteria, resistance of *B. anthracis* to traditional antimicrobials can complicate treatment regimens. New drugs are essential to address these resistant strains, particularly in situations requiring urgent treatment without knowledge of the resistance profile as in a bioterror event [5,6].

Inhibition of the critical metabolic enzyme dihydrofolate reductase (DHFR) is an actively pursued area in antibacterial research, and its value as a target has been validated by the success of the antibiotic trimethoprim (TMP) [7]. New compounds with pharmacokinetics differing from those of TMP are sought to address different sites of infection and then, indirectly, the problem of bacterial resistance. In addition, some bacteria, including *B. anthracis*, encode a chromosomal DHFR that is resistant to TMP but can be targeted by other antifolates, as we have demonstrated previously [8,9,10]. We now have an expanded library of dihydrophthalazine appended 2,4-diaminopyrimidines with demonstrated potency against the DHFR [6] found in *B. anthracis* and other Gram-positive bacteria [11,12,13,14,15,16]. In particular, alteration of the substituent at the C1 stereocenter of the dihydrophthalazine has been demonstrated to modulate interactions at the interface of the protein surface and the surrounding solvent. In our effort to develop a more active drug for *B. anthracis*, our current library presents a refinement of the group at this position to optimize potency against this organism.

## 2. Results and Discussion

### 2.1. Chemistry

**Scheme 1:**
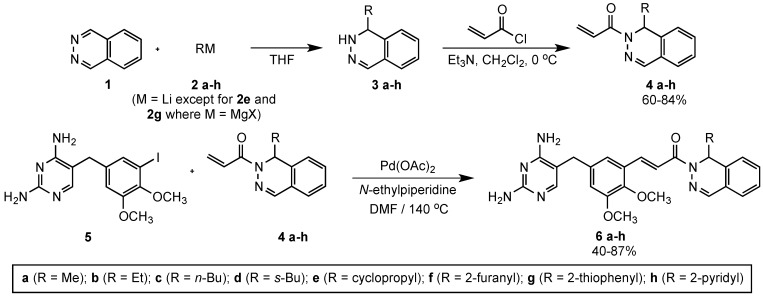
Synthesis of drug candidates **6a**–**h**.

**Scheme 2:**
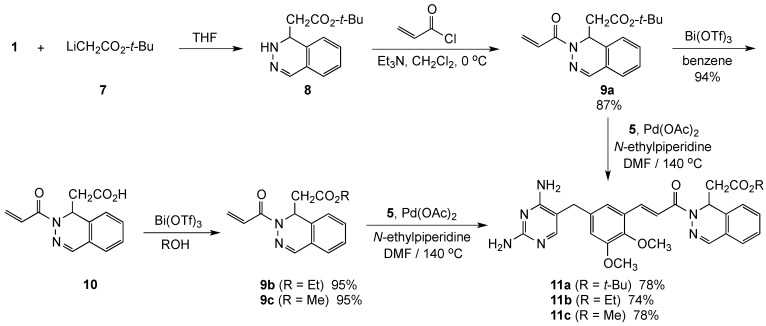
Synthesis of drug candidates **11a**–**c**.

In an effort to develop more active compounds against *B. anthracis* and other Gram-positive bacteria, an earlier synthetic strategy to prepare related structures was modified [14,15]. In this project, we synthesized a series of racemic targets as shown in Scheme 1 and Scheme 2. Starting with commercially available phthalazine (**1**), treatment with an organolithium or organomagnesium reagent (compounds **2a**–**h**) in THF under anhydrous conditions furnished racemic adducts **3a**–**h**. These substrates were further subjected to *N*-acylation using acryloyl chloride and triethylamine to obtain the 1-(phthalazin-2(1*H*)-yl)prop-2-en-1-one derivatives **4a**–**h**. Acrylamides **4a**–**h** were then linked to the known 2,4-diaminopyrimidine intermediate **5** [15] via a Heck coupling in the presence of Pd(OAc)_2_ and *N*-ethylpiperidine to afford targets **6a**–**h** in yields of 40%–87% (Scheme 1) [16,17].

In addition, we have also developed a synthetic route for the preparation of several ester-containing drug candidates (Scheme 2). These targets were assembled by addition of *t*-butyl lithioacetate to **1** to give ester **8**, followed by *N*-acylation with acryloyl chloride to give racemic *t*-butyl 2-(phthalazin-2(1*H*)-yl)acetate (**9a**) in 87% yield. Mild hydrolysis of **9a** using catalytic Bi(OTf)_3_ led to acid **10**, which was re-esterified using this same catalyst in the presence of ethanol or methanol [18] to give **9b** and **9c**, respectively, in 95% yields. Finally, Heck coupling of **9a**–**c** furnished the desired ester-substituted derivatives **11a**–**c** in 74%–78% yields.

### 2.2. Biological Potency

The potency of our synthesized compounds was evaluated in a whole cell model using bacterial cultures, and for activity against the purified DHFR protein target. The ability to halt the growth of standardized cultures gives insight into the utility of the compound as a potential therapeutic, but it does not inform on the cellular target. In the case of whole cells, the lowest concentration of compound needed to inhibit all visible bacterial growth was assessed as in previous studies [9,10,12,15,16] and followed the Clinical Laboratory Standards Institute guidelines [19]. These values are reported in Table 1 as the minimum inhibitory concentration (MIC) in μg/mL. The activity of each compound was evaluated by its ability to halt the enzymatic reaction carried out by the purified DHFR protein in a standardized assay. The results are reported as the compound concentration, in nM, required to inhibit the enzyme activity to one-half the uninhibited rate. This concentration was then used in combination with the substrate affinity of the DHFR enzyme, in this case the K_M_ for dihydrofolate, to derive the inhibition constant K_i_ as reported in Table 1. The combination of the MIC and the K_i_ allowed unbiased assessment of compound potency between bacterial species.

**Table 1:** MIC and K_i_ values of the substrates **6a**–**h** and **11a**–**c** against *B. anthracis.*

Compound	MIC (μg/mL)	K_i_ (nM) ± SEM
	*B. anthracis*	*B anthracis* DHFR
TMP	>128 *	~8,770 *
RAB1	2–4 *	9.4 ± 0.2 *
6a	2.0	8.8 *±* 0.2
6b	1.0	6.8 *±* 0.2
6c	2–4	7.9 *±* 0.2
6d	4	10.4 *±* 0.2
6e	0.5	4.9 *±* 0.2
6f	4	5.9 *±* 0.2
6g	4	8.4 *±* 0.2
6h	4	9.0 *±* 0.2
11a	8	59.0 *±* 0.9
11b	8	24.9 *±* 0.3
11c	8	20.0 *±* 0.3

* Indicates previously published data: TMP [8]; RAB1 [9,10,12,15,16]; K_i_ = 50% inhibition normalized for the intrinsic affinity for the substrate, as outlined in the Cheng-Prusoff formalism [20]. SEM = standard error of the mean. MIC values report the range of values from two independent experiments performed in duplicate. K_i_ values report the mean from at least three independent measurements.

**Figure 1:**
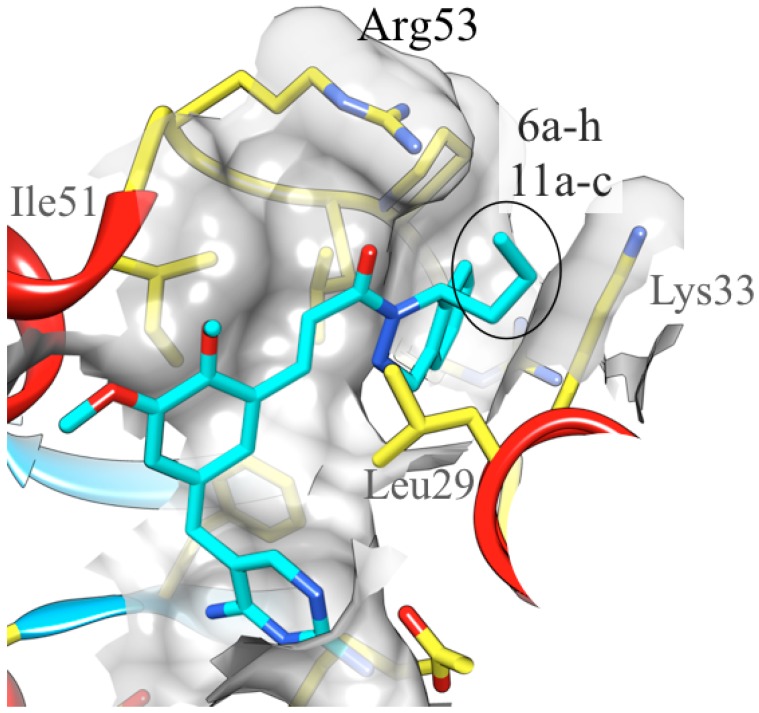
Interactions between the DHFR protein and the RAB1 (R = *n*–Pr) inhibitor. This structure illustrates the position of substituents R at the C1 stereocenter of the dihydrophthalazine with a black oval; selected residues are labeled. It is hypothesized that the superior potency of compound **6e** (R = cyclopropyl) results from stacking interactions with the guanidinium group of Arg 53.

These studies build upon previous results [9,10,12,15,16] and highlight a clear preference for small or planar groups at the C1 dihydrophthalazine stereocenter. Compounds **6a**–**d** are derivatives bearing alkyl substituents at this site, similar to RAB1 (R = *n*–Pr), but with variable lengths. Of these modified derivatives, **6b** (R = Et) showed the greatest activity, while **6a** (R = Me) was intermediate. Derivatives **6c** (R = *n*–Bu) and **6d** (R = *s*–Bu) proved the least efficacious within this series. Placement of heteroaromatic groups and acetic ester moieties at C1 of the dihydrophthalazine, as in **6f**–**h** and **11a**–**c**, respectively, yielded moderately active structures, but these possessed the lowest activities in the current screening. While compounds **11a**–**c** did not demonstrate exceptional potency, the intent was to utilize the ester-bearing modifications as pro-drugs. Within the body, numerous esterase enzymes would carry out cleavage of these esters to generate the acid [21]. It was anticipated that this form would be more soluble in aqueous medium and would be more potent than the parent compound. This, however, was apparently not the case. Furthermore, while we have prepared the acid, we have been unable to purify it to an acceptable level for screening. Finally, the installation of a cyclopropyl group at C1 gave structure **6e**, which is the most potent compound generated to date. Based on available crystallographic studies of RAB1, the cyclopropyl moiety likely forms favorable stacking interactions with an arginine residue at position 53 within the *B. anthracis* DHFR binding site (Figure 1) [9,10].

## 3. Experimental

### 3.1. General Information

Commercial anhydrous *N,N*-dimethylformamide (DMF) and dimethyl sulfoxide (DMSO) were stored under dry N_2_ and transferred by syringe into reactions when required. Tetrahydrofuran (THF) was dried over KOH pellets and distilled from LiAlH_4_ prior to use. K_2_CO_3_ was dried at 120 °C under high vacuum for a period of 16 h before use. All other commercial reagents were used as received. Unless otherwise specified, all reactions were run under dry N_2_ in oven-dried glassware. The saturated NaCl and NH_4_Cl used in workup procedures were aqueous solutions. Reactions were monitored by thin layer chromatography (TLC) on silica gel GF plates (Analtech, 21521). Preparative separations were performed by chromatography on silica gel (Davisil^®^, grade 62, 60–200 mesh) mixed with UV-active phosphor (Sorbent Technologies, UV-05). Band elution for all chromatographic separations was monitored using a hand-held UV lamp. Melting points were uncorrected. FT-IR spectra were run as thin films on NaCl disks. ^1^H- and ^13^C-NMR spectra were measured at 300 MHz or 400 MHz (^1^H) and 75 or 100 MHz (^13^C) in the indicated solvent. Chemical shifts (δ) are referenced to internal (CH_3_)_4_Si and coupling constants (*J*) are given in Hz. Elemental analyses were ±0.4% from Atlantic Microlab, Inc. (Norcross, GA, USA).

### 3.2. Synthesis of 1-(Phthalazin-2(1H)-yl)prop-2-en-1-ones **4a**‒**h**

*(±)-1-(1-Methylphthalazin-2(1H)-yl)prop-2-en-1-one* (**4a**). A stirred solution of phthalazine (**1**) (2.00 g, 15.4 mmol) in dry THF (50 mL) was treated dropwise with a solution of methyllithium (**2a**, 1.5 M in ether, 11.3 mL, 16.9 mmol) over a period of 15 min at –20 °C. The reaction was stirred at this temperature for 45 min and was then poured into saturated NH_4_Cl (50 mL) and extracted with ethyl acetate (3 × 50 mL). The combined organic extracts were washed with saturated NaCl (100 mL), dried (MgSO_4_), filtered, and concentrated under vacuum to afford **3a** as a dark brown liquid. The crude product **3a** was dissolved in dichloromethane (DCM, 50 mL), and triethylamine (1.86 g, 2.56 mL, 18.4 mmol) was added, followed by dropwise addition of acryloyl chloride (1.39 g, 1.25 mL, 15.4 mmol) at 0 °C. The reaction mixture was stirred at 0 °C for 2 h. The reaction was then quenched with saturated NaCl (100 mL), the organic layer was separated, and the aqueous layer was extracted with DCM (2 × 50 mL). The combined organic extracts were washed with saturated NaCl (50 mL), dried (MgSO_4_), filtered, and concentrated to afford the crude product. The crude product was purified on a silica gel column eluted with hexanes:EtOAc (4:1) to afford **4a** as a pale yellow liquid (2.60 g, 84%). IR: 1663, 1621 cm^−1^; ^1^H-NMR (300 MHz, CDCl_3_): δ 7.60 (s, 1H), 7.43 (td, *J* = 7.1, 1.6 Hz, 1H), 7.37–7.23 (complex m, 3H), 7.16 (d, *J* = 7.7 Hz, 1H), 6.49 (dd, *J* = 17.5, 2.2 Hz, 1H), 5.90 (q, *J* = 6.6 Hz, 1H), 5.78 (dd, *J* = 10.4, 2.2 Hz, 1H), 1.31 (d, *J* = 6.6 Hz, 3H); ^13^C-NMR (75 MHz, CDCl_3_): δ 165.8, 141.4, 135.2, 131.5, 128.0, 127.7, 126.9, 125.42, 125.40, 122.9, 47.1, 20.9.

*(±)-1-(1-Ethylphthalazin-2(1H)-yl)prop-2-en-1-one* (**4b**). This compound was prepared as above using **1** (2.00 g, 15.4 mmol) and ethyllithium (**2b**, 1.5 M in dibutyl ether, 11.2 mL, 16.9 mmol), followed by triethylamine (1.86 g, 2.56 mL, 18.4 mmol) and acryloyl chloride (1.39 g, 1.25 mL, 15.4 mmol) to afford **4b** (2.63 g, 80%) as a viscous, colorless oil. IR: 1666, 1621 cm^−1^; ^1^H-NMR (300 MHz, CDCl_3_): δ 7.60 (s, 1H), 7.43 (td, *J* = 7.7, 1.1 Hz, 1H), 7.39–7.28 (complex m, 2H), 7.27 (d, *J* = 7.1 Hz, 1H), 7.14 (d, *J* = 7.1 Hz, 1H), 6.48 (dd, *J* = 17.0, 2.2 Hz, 1H), 5.77 (overlapping dd, *J* = 10.4, 2.2 Hz, 1H and t, *J* = 6.6 Hz, 1H), 1.64 (m, 2H), 0.81 (t, *J* = 7.7 Hz, 3H); ^13^C-NMR (75 MHz, CDCl_3_): δ 166.1, 142.1, 133.4, 131.2, 128.1, 127.9, 127.0, 126.4, 125.5, 123.7, 52.3, 28.0, 9.3.

*(±)-1-(1-n-Butylphthalazin-2(1H)-yl)prop-2-en-1-one* (**4c**). This compound was prepared as above using 1 (2.00 g, 15.4 mmol) and n-butyllithium (2c, 2.2 M in hexanes, 7.68 mL, 16.9 mmol), followed by triethylamine (1.86 g, 2.56 mL, 18.4 mmol) and acryloyl chloride (1.39 g, 1.25 mL, 15.4 mmol) to afford 4c (3.09 g, 83%) as viscous, colorless oil. IR: 1665, 1621 cm^‒1^; ^1^H-NMR (300 MHz, CDCl3): δ 7.62 (s, 1H), 7.44 (td, *J* = 7.7, 1.6 Hz, 1H), 7.35 (td, *J* = 7.1, 1.1 Hz, 1H), 7.32 (dd, *J* = 17.0, 10.4 Hz, 1H), 7.28 (d, *J* = 7.1 Hz, 1H), 7.16 (d, *J* = 7.1 Hz, 1H), 6.48 (dd, *J* = 17.0, 2.2 Hz, 1H), 5.84 (t, *J* = 6.6 Hz, 1H), 5.78 (dd, *J* = 10.4, 2,2 Hz, 1H), 1.64 (q, *J* = 6.6 Hz, 2H), 1.23 (m, 4H), 0.82 (t, *J* = 6.8 Hz, 3H); ^13^C-NMR (75 MHz, CDCl3): δ 166.1, 142.4, 134.0, 131.3, 128.2, 127.9, 127.1, 126.4, 125.6, 123.8, 51.2, 34.8, 26.9, 22.4, 13.8.

*(±)-1-(1-s-Butylphthalazin-2(1H)-yl)prop-2-en-1-one* (**4d**). This compound was prepared as above using **1** (2.00 g, 15.4 mmol) and *s*-butyllithium (2d, 1.4 M in cyclohexane, 12.1 mL, 16.9 mmol), followed by triethylamine (1.86 g, 2.56 mL, 18.4 mmol) and acryloyl chloride (1.39 g, 1.25 mL, 15.4 mmol) to afford 4d (3.00 g, 81%) as a viscous, colorless oil. IR: 1663, 1620 cm^−1^; ^1^H-NMR (300 MHz, CDCl_3_, mixture of diastereomers): δ 7.64 and 7.61 (2s, 1H), 7.44 (t, *J* = 7.7 Hz, 1H), 7.40–7.25 (complex m, 3H), 7.17 (apparent t, *J* = 7.1 Hz, 1H), 6.46 (d, *J* = 17.0 Hz, 1H), 5.76 (m, 2H), 1.73 (m, 1H), 1.46 (m, 1H), 1.10 (m, 1H), 0.92 and 0.82 (2t, *J* = 7.1 Hz, 3H), 0.88 and 0.70 (2d, *J* = 6.6 Hz, 3H); ^13^C-NMR (75 MHz, CDCl_3_, mixture of diastereomers): δ 166.5, 143.4, 143.1, 132.5, 131.6, 131.1, 131.0, 128.2, 127.95, 127.90, 127.5, 127.4, 127.2, 125.4, 124.7, 124.4, 55.74, 55.26, 40.6, 39.9, 25.4, 24.3, 15.0, 14.2, 11.6, 11.4.

*(±)-1-(1-Cyclopropylphthalazin-2(1H)-yl)prop-2-en-1-one* (**4e**). To a stirred solution of **1** (2.00 g, 15.4 mmol) in dry THF (50 mL) was added dropwise cyclopropylmagnesium chloride (0.5 M in THF, 33.8 mL, 16.9 mmol) over a period of 10 min at 0 °C. The reaction was stirred at 0 °C for 2 h and was then quenched with saturated NH_4_Cl (50 mL) and extracted with ethyl acetate (2 × 50 mL). The combined extracts were washed with saturated NaCl, dried (MgSO_4_), filtered, and concentrated to give **3e** as a light brown oil. The crude product **3e** was acylated as described for compound **4a** using triethylamine (1.86 g, 2.56 mL, 18.4 mmol) and acryloyl chloride (1.39 g, 1.25 mL, 15.4 mmol) in DCM (50 mL) to obtain **4e** (2.71 g, 78%) as a pale yellow oil. IR: 1662, 1621 cm^−1^; ^1^H-NMR (300 MHz, DMSO-*d*_6_): δ 7.66 (s, 1H), 7.45 (td, *J* = 7.1, 1.1 Hz, 1H), 7.37 (td, *J* = 7.7, 1.1 Hz, 1H), 7.36 (dd, *J* = 17.0, 10.4 Hz, 1H), 7.30 (m, 2H), 7.16 (d, *J* = 7.7 Hz, 1H), 6.48 (dd, *J* = 17.0, 2.2 Hz, 1H), 5.79 (dd, *J* = 10.4, 2.2 Hz, 1H), 5.47 (d, *J* = 7.7 Hz, 1H), 1.17 (m, 1H), 0.66 (quintet, *J* = 4.9 Hz, 1H), 0.44 (m, 1H), 0.36 (m, 1H); ^13^C-NMR (100 MHz, CDCl_3_): δ 166.6, 142.5, 132.9, 131.5, 128.3, 128.1, 127.2, 126.5, 125.5, 124.0, 54.0, 16.7, 3.8, 2.3.

*(±)-1-(1-(Furan-2-yl)phthalazin-2(1H)-yl)prop-2-en-1-one* (**4f**). To a stirred solution of furan (1.20 g, 17.6 mmol) in dry THF (20 mL) was added dropwise *n*-butyllithium (2.5 M in hexanes, 7.30 mL, 18.3 mmol) over a period of 30 min at –78 °C. The solution was warmed to –25 °C, and stirring was continued at this temperature for 30 min. The reaction mixture was cooled back to –78 °C, and a solution of 1 (2.00 g, 15.3 mmol) in dry THF (20 mL) was added dropwise over 30 min. The reaction mixture was stirred at this temperature for 2 h. The mixture was poured into saturated NH_4_Cl (100 mL) and extracted with ethyl acetate (3 × 50 mL). The combined organic extracts were then washed with saturated NaCl (50 mL), dried (MgSO_4_), filtered, and concentrated under vacuum to afford 3f as a light brown oil. The crude product **3f** was dissolved in DCM (30 mL), and triethylamine (2.37 g, 3.26 mL, 23.4 mmol) was added, followed by dropwise addition of acryloyl chloride (1.59 g, 1.43 mL, 17.6 mmol) at 0 °C. The reaction mixture was stirred at 0 °C for 2 h. The reaction was then quenched with saturated NaCl (25 mL), and the organic layer was separated. The aqueous layer was extracted with DCM (2 × 30 mL), and the combined organic extracts were washed with saturated NaCl (50 mL), dried (MgSO_4_), filtered, and concentrated to afford the crude product. The product was purified on a silica gel column eluted with hexanes–EtOAc (7:3) to afford **4f** (2.66 g, 60%) as a yellow liquid. IR: 1666, 1616 cm^−1^; ^1^H-NMR (400 MHz, CDCl_3_): δ 7.68 (s, 1H), 7.47 (dd, *J* = 7.4, 1.4 Hz, 1H), 7.41 (td, *J* = 7.4, 1.2 Hz, 1H), 7.37–7.26 (complex m, 3H), 7.25 (d, *J* = 1.8 Hz, 1H), 7.04 (s, 1H), 6.52 (dd, *J* = 17.1, 2.0 Hz, 1H), 6.19 (dd, *J* = 3.2, 1.8 Hz, 1H), 5.94 (d, *J* = 3.2 Hz, 1H), 5.80 (dd, *J* = 10.5, 2.0 Hz, 1H); ^13^C-NMR (100 MHz, CDCl_3_): δ 166.2, 149.5, 143.5, 142.0, 131.5, 131.2, 128.7, 128.0, 126.6, 126.1, 126.0, 123.7, 111.7, 111.3, 41.8.

*(±)-1-(1-(Thiophen-2-yl)phthalazin-2(1H)-yl)prop-2-en-1-one* (**4g**). This compound was prepared by addition of 2-thiophenylmagnesium bromide, prepared from 2-bromothiophene (1.77 g, 1.69 mL, 21.0 mmol) and magnesium (0.69 g, 28.4 mmol) in dry THF (25 mL), to a solution of **1** (2.50 g, 19.2 mmol) in dry THF (30 mL). Product **3g** was acylated using triethylamine (2.80 g, 3.86 mL, 27.7 mmol) and acryloyl chloride (1.90 g, 1.71 mL, 21.0 mmol) in DCM to afford 4g (3.09 g, 60%) as a light yellow liquid. IR: 1662, 1617 cm^−1^; ^1^H-NMR (400 MHz, CDCl_3_): δ 7.68 (d, *J* = 7.6 Hz, 1H), 7.48 (td, *J* = 7.6, 1.2 Hz, 1H), 7.46-7.32 (complex m, 4H), 7.29 (d, *J* = 7.4 Hz, 1H), 7.13 (dd, *J* = 5.1, 3.7 Hz, 1H), 6.51 (dd, *J* = 17.1, 2.0 Hz, 1H), 5.83 (dd, *J* = 10.3, 2.0 Hz, 1H), 5.02 (s, 2H); ^13^C-NMR (100 MHz, CDCl_3_): δ 166.2, 143.7, 141.8, 132.1, 131.9, 129.1, 128.7, 127.0, 126.8, 126.3, 126.2, 125.84, 125.80, 123.7, 49.1.

*(±)-1-(1-(Pyridin-2-yl)phthalazin-2(1H)-yl)prop-2-en-1-one* (**4h**). This compound was prepared by the procedure described for **4f** using 2-bromopyridine (1.30 g, 8.22 mmol), *n*-butyllithium (2.5 M in hexanes, 3.38 mL, 8.45 mmol), and **1** (1.00 g, 7.69 mmol) in dry THF (25 mL). Product **3h** was acylated using triethylamine (0.93 g, 1.28 mL, 9.2 mmol) and acryloyl chloride (0.70 g, 0.63 mL, 7.73 mmol) in DCM (30 mL) to afford **4h** (1.53 g, 76%) as a light yellow liquid. IR: 1662, 1617 cm^−1^; ^1^H-NMR (400 MHz, CDCl_3_): δ 8.48 (dq, *J* = 4.9, 0.8 Hz, 1H), 7.63 (s, 1H), 7.57 (td, *J* = 7.6, 1.8 Hz, 1H), 7.47–7.37 (complex m, 3H), 7.34 (td, *J* = 7.6, 1.8 Hz, 1H), 7.28 (d, *J* = 7.8 Hz, 2H), 7.08 (ddd, *J* = 7.6, 4.9, 1.0 Hz, 1H), 6.94 (s, 1H), 6.47 (dd, *J* = 17.4, 2.0 Hz, 1H), 5.80 (dd, *J* = 10.3, 2.0 Hz, 1H); ^13^C-NMR (100 MHz, CDCl_3_): δ 166.6, 159.6, 149.7, 141.2, 136.8, 132.2, 131.8, 129.0, 128.5, 127.5, 127.1, 126.1, 122.5, 122.2, 120.3, 56.7.

### 3.3. Synthesis of 2,4-Diaminopyrimidine **5**

*2,4-Diamino-5(5-iodo-3,4-dimethoxybenzyl)pyrimidine* (**5**). This compound was prepared in 60% yield from morpholine and acrylonitrile on a 0.47-mol scale according to the literature procedure [15], mp 217–218 °C (lit. [15] mp 217–218 °C). IR: 3467, 3315, 3140, 1638 cm-1; ^1^H-NMR (300 MHz, DMSO-*d*_6_): δ 7.57 (s, 1H), 7.14 (d, *J* = 1.8 Hz, 1H), 6.98 (d, *J* = 1.8 Hz, 1H), 6.16 (br s, 2H), 5.77 (br s, 2H), 3.77 (s, 3H), 3.66 (s, 3H), 3.54 (s, 2H); ^13^C-NMR (75 MHz, DMSO-*d*_6_): δ 162.4, 162.1, 156.0, 152.0, 146.3, 138.9, 129.1, 113.8, 105.2, 92.4, 59.8, 55.8, 31.7.

### 3.4. Synthesis of Drug Candidates **6**

*(±)-(E)-3-(5-((2,4-Diaminopyrimidin-5-yl)methyl)-2,3-dimethoxyphenyl)-1-(1-methylphthalazin-2(1H)-yl)prop-2-en-1-one* (**6a**). To a stirred solution of **5** (1.00 g, 2.59 mmol) in dry DMF (6 mL) was added a solution of 4a (0.57 g, 2.85 mmol) in DMF (2 mL), followed by *N*-ethylpiperidine (0.32 g, 0.40 mL, 2.84 mmol) and Pd(OAc)_2_ (20 mg, 0.089 mmol). The reaction was heated at 140 °C for 20 h and then cooled using an ice bath. The product was purified by directly pouring the crude reaction mixture onto a 50 cm × 2.5 cm silica gel chromatography column slurry packed with CH_2_Cl_2_. Impurities were eluted using CH_2_Cl_2_, and the final product was collected using CH_2_Cl_2_/MeOH/Et_3_N (97:3:1). Evaporation of the solvent gave an oily, yellow-brown foam, which was dried under high vacuum for a period of 2 h. Methanol (3 mL) was added, followed by ether (10 mL), to crystallize the product, and the mixture was allowed to cool for 4 h. The product was filtered and dried under vacuum to afford **6a** (0.97 g, 82%) as an off-white solid, mp 153–155 °C. IR: 3612, 3308, 3192, 1634, 1600 cm^−1^; ^1^H-NMR (400 MHz, DMSO-*d*_6_): δ 7.93 (s, 1H), 7.88 (d, *J* = 16.5 Hz, 1H), 7.64 (d, *J* = 16.5 Hz, 1H), 7.62–7.35 (complex m, 5H), 7.29 (s, 1H), 7.15 (br s, 2H), 7.04 (s, 1H), 6.64 (br s, 2H), 5.89 (m, 1H), 3.81 (s, 3H), 3.76 (s, 3H), 3.65 (s, 2H), 1.23 (d, *J* = 6.0 Hz, 3H); ^13^C-NMR (100 MHz, DMSO-*d*_6_): δ 165.5, 163.0, 158.6, 152.6, 148.6, 146.2, 142.1, 136.6, 135.3, 135.1, 132.0, 128.2, 127.9, 126.1, 126.0, 123.0, 118.7, 118.0, 114.9, 107.2, 60.8, 55.8, 46.8, 32.0, 21.1. Anal. Calcd for C_25_H_26_N_6_O_3_·3.9 H_2_O: C, 56.79; H, 6.44; N, 15.89. Found: C, 56.75; H, 6.32; N, 15.63.

*(±)-(E)-3-(5-((2,4-Diaminopyrimidin-5-yl)methyl)-2,3-dimethoxyphenyl)-1-(1-ethylphthalazin-2(1H)-yl)prop-2-en-1-one* (**6b**). This compound was prepared as above using **5** (1.00 g, 2.59 mmol), **4b** (0.61 g, 2.85 mmol), *N*-ethylpiperidine (0.32 g, 0.40 mL, 2.85 mmol), and Pd(OAc)_2_ (20 mg, 0.089 mmol) in dry DMF (8 mL) to give **6b** (0.98 g, 80%) as an off-white solid, mp 232–234 °C. IR: 3472, 3325, 3179, 1635, 1598 cm^−1^; ^1^H-NMR (400 MHz, DMSO-*d*_6_): δ 7.92 (s, 1H), 7.88 (d, *J* = 15.9 Hz, 1H), 7.65 (d, *J* = 15.9 Hz, 1H), 7.62 (s, 1H), 7.58-7.43 (complex m, 3H), 7.40 (d, *J* = 7.1 Hz, 1H), 7.26 (s, 1H), 7.00 (s, 1H), 6.21 (br s, 2H), 5.80 (t, *J* = 6.6 Hz, 1H), 5.77 (br s, 2H), 3.80 (s, 3H), 3.74 (s, 3H), 3.61 (s, 2H), 1.61 (m, 2H), 0.74 (t, *J* = 7.7 Hz, 3H); ^13^C-NMR (100 MHz, DMSO-*d*_6_): δ 165.7, 162.4, 162.2, 155.9, 152.5, 146.0, 142.6, 136.7, 133.2, 131.7, 128.3, 127.8, 126.7, 126.1, 123.7, 118.4, 117.9, 114.7, 105.8, 60.8, 55.8, 51.7, 32.5, 27.8, 9.3 (1 aromatic C unresolved). Anal. Calcd for C_26_H_28_N_6_O_3_: C, 65.83; H, 5.99; N, 17.72. Found: C, 65.84; H, 5.96; N, 17.63.

*(±)-(E)-1-(1-n-Butylphthalazin-2(1H)-yl)-3-(5-((2,4-diaminopyrimidin-5-yl)methyl)-2,3-dimethoxyphenyl)prop-2-en-1-one* (**6c**). This compound was prepared as above using **5** (1.00 g, 2.59 mmol), **4c** (0.69 g, 2.85 mmol), *N*-ethylpiperidine (0.32 g, 0.40 mL, 2.85 mmol), and Pd(OAc)_2_ (20 mg, 0.089 mmol) in dry DMF (8 mL) to give **6c** (0.97 g, 75%) as an off-white solid, mp 112–114 °C. IR: 3474, 3329, 3177, 1637, 1603 cm^−1^; ^1^H-NMR (400 MHz, DMSO-*d*_6_): δ 7.94 (s, 1H), 7.87 (d, *J* = 15.9 Hz, 1H), 7.63 (d, *J* = 15.9 Hz, 1H), 7.61 (s, 1H), 7.52 (m, 2H), 7.45 (d, *J* = 7.1 Hz, 1H), 7.39 (d, *J* = 7.1 Hz, 1H), 7.25 (s, 1H), 7.00 (s, 1H), 6.22 (br s, 2H), 5.84 (t, *J* = 6.6 Hz, 1H), 5.78 (br s, 2H), 3.80 (s, 3H), 3.74 (s, 3H), 3.60 (s, 2H), 1.58 (m, 2H), 1.17 (m, 4H), 0.79 (t, *J* = 6.6 Hz, 3H); ^13^C-NMR (100 MHz, DMSO-*d_6_*): δ 165.6, 162.22, 162.18, 155.6, 152.5, 146.0, 142.8, 136.6 (2C), 133.6, 131.7, 128.2, 127.8, 126.5, 126.0, 123.6, 118.3, 117.8, 114.7, 105.8, 60.8, 55.7, 50.5, 34.3, 32.4, 26.6, 21.9, 13.8. Anal. Calcd for C_28_H_32_N_6_O_3_·0.8 H_2_O: C, 65.30; H, 6.58; N, 16.32. Found: C, 65.24; H, 6.39; N, 16.22.

*(±)-(E)-1-(1-s-Butylphthalazin-2(1H)-yl)-3-(5-((2,4-diaminopyrimidin-5-yl)methyl)-2,3-dimethoxyphenyl)prop-2-en-1-one* (**6d**). This compound was prepared as above using **5** (1.00 g, 2.59 mmol), **4d** (0.69 g, 2.85 mmol), *N*-ethylpiperidine (0.32 g, 0.40 mL, 2.85 mmol), and Pd(OAc)_2_ (20 mg, 0.089 mmol) in dry DMF (8 mL) to give **6d** (0.93 g, 72%) as an off-white solid, mp 122–124 °C. IR: 3469, 3371, 3214, 1634, 1603 cm^−1^; ^1^H-NMR (400 MHz, DMSO-*d*_6_, mixture of diastereomers): δ 7.97 and 7.95 (2s, 1H), 7.86 (d, *J* = 15.9 Hz, 1H), 7.66 (2d, *J* = 15.9 Hz, 1H), 7.60 (s, 1H), 7.59-7.42 (complex m, 3H), 7.37 (apparent t, *J* = 7.7 Hz, 1H), 7.27 (s, 1H), 7.00 (s, 1H), 6.32 (br s, 2H), 5.86 (br s, 2H), 5.74 (d, *J* = 6.6 Hz, 1H), 3.79 (s, 3H), 3.74 (s, 3H), 3.60 (s, 2H), 1.64 (m, 1H), 1.39 (m, 1H), 0.94 (m, 1H), 0.87 and 0.78 (2t, *J* = 7.1 Hz, 3H), 0.81 and 0.65 (2d, *J* = 7. 1 Hz, 3H); ^13^C-NMR (100 MHz, DMSO-*d*_6_, mixture of diastereomers) δ 165.9, 165.6, 162.3, 161.8, 154.8, 152.5, 146.0, 143.8, 143.5, 136.6, 136.4, 132.0, 131.4, 131.1, 128.3, 127.8, 127.5, 127.4, 125.9, 124.4, 124.2, 118.4, 117.9, 114.7, 105.9, 60.8, 55.7, 54.5, 32.4, 24.9, 24.0, 15.0, 14.2, 11.4, 11.3. *Anal*. Calcd for C_28_H_32_N_6_O_3_·1.0 H_2_O: C, 64.85; H, 6.61; N, 16.20. Found: C, 64.83; H, 6.43; N, 16.28.

*(±)-(E)-1-(1-Cyclopropylphthalazin-2(1H)-yl)-3-(5-((2,4-diaminopyrimidin-5-yl)methyl)-2,3-dimethoxyphenyl)prop-2-en-1-one* (**6e**). This compound was prepared as above using **5** (1.00 g, 2.59 mmol), **4e** (0.64 g, 2.85 mmol), *N*-ethylpiperidine (0.32 g, 0.40 mL, 2.85 mmol), and Pd(OAc)_2_ (20 mg, 0.089 mmol) in dry DMF (8 mL) to give **6e** (0.90 g, 72%) as an off-white solid, mp 155–157 °C. IR: 3464, 3359, 3202, 1636, 1602 cm^−1^; ^1^H-NMR (400 MHz, DMSO-*d*_6_): δ 7.99 (s, 1H), 7.87 (d, *J* = 15.9 Hz, 1H), 7.66 (d, *J* = 15.9 Hz, 1H), 7.59 (s, 1H), 7.53 (d, *J* = 7.1 Hz, 2H), 7.46 (t, *J* = 7.1 Hz, 2H), 7.28 (s, 1H), 7.02 (s, 1H), 6.64 (br s, 2H), 6.16 (br s, 2H), 5.42 (d, *J* = 8.8 Hz, 1H), 3.80 (s, 3H), 3.75 (s, 3H), 3.62 (s, 2H), 1.13 (m, 1H), 0.55 (m, 1H), 0.44 (m, 2H), 0.33 (m, 1H); ^13^C-NMR (100 MHz, DMSO-*d*_6_): δ 165.9, 162.6, 160.4, 152.5, 152.1, 146.1, 142.9, 136.7, 135.9, 132.8, 131.8, 128.3, 127.8, 126.6, 125.9, 123.7, 118.6, 118.1, 114.8, 106.5, 60.8, 55.8, 53.4, 32.2, 16.7, 4.0, 2.0. Anal. Calcd for C_27_H_28_N_6_O_3_·2.2 H_2_O: C, 61.87; H, 6.23; N, 16.03. Found: C, 61.88; H, 6.32; N, 16.00.

*(±)-(E)-3-(5-((2,4-Diaminopyrimidin-5-yl)methyl)-2,3-dimethoxyphenyl)-1-(1-(furan-2-yl)phthalazin-2(1H)-yl)prop-2-en-1-one* (**6f**). This compound was prepared as above using **5** (1.50 g, 3.88 mmol), **4f** (1.05 g, 4.15 mmol), *N*-ethylpiperidine (0.48 g, 0.58 mL, 4.27 mmol), and Pd(OAc)_2_ (30 mg, 0.134 mmol) in dry DMF (10 mL) to give **6f** (0.82 g, 42%), as a brown solid, mp 242–244 °C. IR: 3439, 3336, 3181, 1639, 1601 cm^−1^; ^1^H-NMR (400 MHz, DMSO-*d*_6_): δ 8.01 (s, 1H), 7.91 (d, *J* = 16.0 Hz, 1H), 7.66 (d, *J* = 16.0 Hz, 1H), 7.61-7.49 (complex m, 6H), 7.32 (s, 1H), 7.17 (br s, 2H), 7.09 (s, 1H), 7.05 (s, 1H), 6.67 (br s, 2H), 6.33 (m, 1H), 5.99 (dd, *J* = 3.1, 0.6 Hz, 1H), 3.81 (s, 3H), 3.76 (s, 3H), 3.65 (s, 2H); ^13^C-NMR (100 MHz, DMSO-*d*_6_): δ 165.6, 163.1, 158.1, 152.59, 152.56, 147.8, 146.3, 143.0, 142.4, 137.2, 135.1, 132.1, 130.2, 129.0, 127.8, 127.2, 126.3, 123.7, 118.8, 117.7, 115.1, 110.4, 107.6, 107.4, 60.8, 55.8, 47.4, 32.0. Anal. Calcd for C_28_H_26_N_6_O_4_·4.6 H_2_O·0.1 Et_2_O: C, 56.77; H, 6.07; N, 13.99. Found: C, 56.44; H, 5.85; N, 13.72.

*(±)-(E)-3-(5-((2,4-Diaminopyrimidin-5-yl)methyl)-2,3-dimethoxyphenyl)-1-(1-(thiophen-2-yl)-phthalazin-2(1H)-yl)prop-2-en-1-one (***6g***)*. This compound was prepared as above using **5** (1.00 g, 2.59 mmol), **4g** (0.77 g, 2.85 mmol), *N*-ethylpiperidine (0.32 g, 0.40 mL, 2.85 mmol), and Pd(OAc)_2_ (20 mg, 0.089 mmol) in dry DMF (8 mL) to give **6g** (0.93 g, 68%) as a brown solid, mp 235–237 °C. IR: 3452, 3345, 3179, 1637, 1602 cm^−1^; ^1^H-NMR (400 MHz, DMSO-*d*_6_): δ 8.06 (s, 1H), 7.94 (d, *J* = 16.0 Hz, 1H), 7.70–7.50 (complex m, 6H), 7.40 (d, *J* = 4.1 Hz, 1H), 7.31 (s, 1H), 7.27 (s, 1H), 7.04 (s, 1H), 6.96 (br s, 2H), 6.89 (s, 1H), 6.66 (s, 1H), 6.47 (br s, 2H), 3.81 (s, 3H), 3.77 (s, 3H), 3.64 (s, 2H); ^13^C-NMR (100 MHz, DMSO-*d*_6_): δ 165.7, 162.9, 159.0, 152.6, 149.4, 146.3, 143.8, 142.4, 137.4, 135.4, 132.2, 132.1, 129.0, 127.7, 127.1, 126.5, 126.32, 126.27, 126.0, 123.4, 118.8, 117.7, 115.1, 107.9, 60.8, 55.8, 48.8, 32.1. Anal. Calcd for C_28_H_26_N_6_O_3_S·4.3 H_2_O: C, 55.67; H, 5.77; N, 13.91. Found: C, 55.99; H, 5.75; N, 13.82.

*(±)-(E)-3-(5-((2,4-Diaminopyrimidin-5-yl)methyl)-2,3-dimethoxyphenyl)-1-(1-(pyridin-2-yl)phthalazin-2(1H)-yl)prop-2-en-1-one* (**6h**). This compound was prepared as above using **5** (1.50 g, 3.88 mmol), **4h** (1.10 g, 4.18 mmol), *N*-ethylpiperidine (0.48 g, 0.58 mL, 4.27 mmol), and Pd(OAc)_2_ (30 mg, 0.134 mmol) in dry DMF (10 mL) to give **6h** (0.80 g, 40%) as a brown solid, mp 177–179 °C. IR: 3459, 3347, 3216, 1648, 1611 cm^−1^; ^1^H-NMR (400 MHz, DMSO-*d*_6_): δ 8.43 (d, *J* = 4.1 Hz, 1H), 7.89 (s, 1H), 7.85 (d, *J* = 16.0 Hz, 1H), 7.76 (d, *J* = 16.0 Hz, 1H), 7.74 (td, *J* = 7.4, 1.6 Hz, 1H), 7.61 (m, 2H), 7.50 (m, 2H), 7.42 (m, 2H), 7.30 (s, 1H), 7.23 (dd, *J* = 6.7, 5.0 Hz, 1H), 7.01 (d, *J* = 1.4 Hz, 1H), 6.95 (s, 1H), 6.48 (br s, 2H), 6.01 (br s, 2H), 3.79 (s, 3H), 3.73 (s, 3H), 3.63 (s, 2H); ^13^C-NMR (100 MHz, DMSO-*d*_6_): δ 166.0, 162.4, 161.1, 159.6, 153.5, 152.5, 149.3, 146.1, 140.9, 137.1, 136.9, 136.2, 132.0, 131.8, 128.6, 127.7, 127.5, 126.3, 122.9, 122.8, 120.2, 118.4, 117.8, 114.9, 106.2, 60.8, 56.0, 55.8, 32.3. Anal. Calcd for C_29_H_27_N_7_O_3_·2.4 H_2_O: C, 61.67; H, 5.68; N, 17.36. Found: C, 61.58; H, 5.47; N, 17.43.

### 3.5. Synthesis of Esters **9a**–**c**

*(±)-t-Butyl 2-(2-Acryloylphthalazin-2(1H)-yl)acetate* (**9a**). To a stirred solution of *tert*-butyl acetate (2.67 g, 3.08 mL, 23.0 mmol) in dry THF (40 mL) at –78 °C was added dropwise *n*-butyllithium (2.5 M in hexanes, 7.68 mL, 19.2 mmol) over a period of 30 min. The solution was warmed to –25 °C and stirred at this temperature for a period of 30 min. To this reaction mixture was added a solution of **1** (2.00 g, 15.4 mmol) in dry THF (25 mL), and stirring was continued for an additional 30 min at 0 °C. The reaction mixture was poured into saturated NH_4_Cl (100 mL) and extracted with EtOAc (3 × 50 mL). The organic extracts were washed with saturated NaCl (50 mL), dried (MgSO_4_) and concentrated to afford **8** as a light brown oil. The crude product **8** was then dissolved in DCM (50 mL), and triethylamine (1.86 g, 2.56 mL, 18.4 mmol) was added, followed by dropwise addition of acryloyl chloride (1.39 g, 1.25 mL, 15.4 mmol) at 0 °C. The reaction mixture was stirred at 0 °C for a period of 2 h. The reaction was quenched with saturated NaCl (50 mL), and the organic layer was separated. The aqueous layer was extracted with DCM (2 × 50 mL) and the combined organic layers were washed with saturated NaCl (50 mL), dried (MgSO_4_), filtered, and concentrated to afford the crude product. The crude product was purified on a silica gel column eluted with hexanes–EtOAc (4:1) to afford **9a** (4.00 g, 87%) as a colorless liquid. IR: 1727, 1668, 1621 cm^−1^; ^1^H-NMR (300 MHz, CDCl_3_): δ 8.64 (s, 1H), 7.44 (td, *J* = 7.7 Hz, 1H), 7.38 (td, *J* = 7.7, 1.6 Hz, 1H), 7.36-7.26 (complex m, 3H), 6.49 (dd, *J* = 17.0, 2.2 Hz, 1H), 6.25 (t, *J* = 7.1 Hz, 1H), 5.79 (dd, *J* = 10.4, 2.2 Hz, 1H), 2.58 (d, *J* = 7.1 Hz, 2H), 1.37 (s, 9H); ^13^C-NMR (75 MHz, CDCl_3_): δ 168.7, 166.0, 142.0, 132.5, 131.6, 128.7, 128.4, 126.9, 126.7, 125.7, 123.6, 80.9, 48.0, 40.7, 27.8.

*(±)-2-(2-Acryloylphthalazin-2(1H)-yl)acetic Acid* (**10**). To a stirred solution of **9a** (1.50 g, 5.00 mmol) in benzene (25 mL) was added Bi(OTf)_3_ (0.164 g, 0.25 mmol, 5 mol%), and the solution was refluxed for a period of 2 h. To this solution was added EtOAc (50 mL), followed by H_2_O (50 mL). The organic layer was washed with saturated NaCl (50 mL), dried (MgSO_4_), filtered, and concentrated to afford **10** (1.15 g, 94%) as a pale yellow solid, mp 142–145 °C. IR: 3400, 1734, 1670 cm^−1^; ^1^H-NMR (400 MHz, DMSO-*d*_6_): δ 12.4 (s, 1H), 7.93 (s, 1H), 7.56–7.43 (complex m, 3H), 7.39 (d, *J* = 7.4 Hz, 1H), 7.24 (dd, *J* = 17.2, 10.5 Hz, 1H), 6.34 (dd, *J* = 17.2, 2.1 Hz, 1H), 6.13 (t, *J* = 6.8 Hz, 1H), 5.86 (dd, *J* = 10.5, 2.1 Hz, 1H), 2.51 (m, 2H); ^13^C-NMR (100 MHz, DMSO-*d*_6_): δ 170.7, 165.1, 142.8, 132.3, 131.9, 128.9, 128.7, 127.0, 126.4, 126.3, 123.4, 47.5, 39.5. Attempts to further purify this compound failed to yield material with sufficient purity for biological testing.

*(±)-Ethyl 2-(2-Acryloylphthalazin-2(1H)-yl)acetate* (**9b**). To a stirred solution of **10** (1.00 g, 4.10 mmol) in ethanol (25 mL) was added Bi(OTf)_3_ (0.134 g, 0.20 mmol, 5 mol%), and the mixture was refluxed for a period of 2 h. The solution was concentrated and purified using a silica gel column eluted with hexanes–EtOAc (4:1) to afford **9b** (1.06 g, 95%) as a colorless, viscous liquid. IR: 1733, 1667, 1621 cm^−1^; ^1^H-NMR (300 MHz, CDCl_3_): δ 7.66 (s, 1H), 7.45 (td, *J* = 7.1, 1.1 Hz, 1H), 7.38 (td, *J* = 7.7, 1.1 Hz, 1H), 7.35–7.23 (complex m, 3H), 6.49 (dd, *J* = 17.0, 2.2 Hz, 1H), 6.29 (t, *J* = 6.6 Hz, 1H), 5.81 (dd, *J* = 10.4, 1.6 Hz, 1H), 4.06 (q, *J* = 7.1 Hz, 2H), 2.65 (m, 2H), 1.19 (t, *J* = 7.1 Hz, 3H); ^13^C-NMR (75 MHz, CDCl_3_): δ 169.5, 166.1, 142.1, 132.3, 131.7, 128.9, 128.6, 126.8, 126.6, 125.8, 123.6, 60.7, 47.9, 39.5, 14.0.

*(±)-Methyl 2-(2-Acryloylphthalazin-2(1H)-yl)acetate* (**9c**). To a stirred solution of **10** (1.00 g, 4.10 mmol) in methanol (25 mL) was added Bi(OTf)_3_ (0.134 g, 0.20 mmol, 5 mol%), and the reaction was refluxed for 2 h. The solution was concentrated and purified using a silica gel column eluted with hexanes:EtOAc (4:1) to afford **9c** (1.00 g, 95%) as colorless, viscous liquid. IR: 1732, 1663, 1618 cm^−1^; ^1^H-NMR (300 MHz, CDCl_3_): δ 7.67 (s, 1H), 7.45 (td, *J* = 7.7, 1.6 Hz, 1H), 7.38 (td, *J* = 7.7, 1.6 Hz, 1H), 7.34–7.27 (complex m, 3H), 6.49 (dd, *J* = 17.0, 2.2 Hz, 1H), 6.28 (t, *J* = 7.7 Hz, 1H), 5.81 (dd, *J* = 10.4, 1.6 Hz, 1H), 3.61 (s, 3H), 2.66 (m, 2H); ^13^C-NMR (75 MHz, CDCl_3_): δ 169.9, 166.1, 142.1, 132.2, 131.8, 128.9, 128.6, 126.7, 126.4, 125.8, 123.4, 51.7, 47.9, 39.2.

### 3.6. Synthesis of Drug Candidates **11a**–**c**

*t-Butyl (±)-(E)-2-(2-(3-(5-((2,4-Diaminopyrimidin-5-yl)methyl)-2,3-dimethoxyphenyl)acryloyl)-phthal-azin-2(1H)-yl)acetate* (**11a**). This compound was prepared as described for **6a** using **5** (1.00 g, 2.59 mmol), **9a** (0.86 g, 2.85 mmol), *N*-ethylpiperidine (0.32 g, 0.40 mL, 2.85 mmol), and Pd(OAc)_2_ (20 mg, 0.089 mmol) in dry DMF (8 mL) to give **11a** (1.12 g, 78%) as an off-white solid, mp 185–187 °C. IR: 3361, 3187, 3068, 1698, 1672, 1638 cm^−1^; ^1^H-NMR (400 MHz, DMSO-*d*_6_): δ 7.96 (s, 1H), 7.89 (d, *J* = 15.9 Hz, 1H), 7.64 (d, *J* = 15.9 Hz, 1H), 7.62–7.46 (complex m, 6H), 7.41 (d, *J* = 7.1 Hz, 1H), 7.32 (s, 1H), 7.06 (s, 1H), 7.02 (br s, 2H), 6.18 (t, *J* = 6.6 Hz, 1H), 3.82 (s, 3H), 3.75 (s, 3H), 3.66 (s, 2H), 2.50 (m, 2H), 1.30 (s, 9H); ^13^C-NMR (100 MHz, DMSO-d_6_): δ 168.4, 163.54, 163.46, 156.4, 152.6, 146.3, 144.5, 142.5, 136.8, 134.6, 132.1, 131.9, 128.7, 127.9, 126.5, 126.2, 123.5, 118.8, 117.9, 115.1, 108.0, 80.4, 60.8, 55.9, 47.9, 40.7, 31.8, 27.5. Anal. Calcd for C_30_H_34_N_6_O_5_·6.2 H_2_O: C, 53.75; H, 5.90; N, 12.54. Found: C, 53.71; H, 5.53; N, 12.56.

*Ethyl (±)-(E)-2-(2-(3-(5-((2,4-Diaminopyrimidin-5-yl)methyl)-(2,3-dimethoxyphenyl)acryloyl)-phthal-azin-2(1H)-yl)acetate* (**11b**). This compound was prepared as above using **5** (1.00 g, 2.59 mmol), **9b** (0.78 g, 2.85 mmol), *N*-ethylpiperidine (0.32 g, 0.40 mL, 2.85 mmol), and Pd(OAc)_2_ (20 mg, 0.089 mmol) in dry DMF (8 mL) to give **11b** (1.01 g, 74%) as a pale yellow solid, mp 113–115 °C. IR: 3473, 3352, 3185, 1728, 1651, 1614 cm^−1^; ^1^H-NMR (400 MHz, DMSO-*d*_6_): δ 7.98 (s, 1H), 7.88 (d, *J* = 15.9 Hz, 1H), 7.61 (d, *J* = 15.9 Hz, 1H), 7.60 (s, 1H), 7.52 (complex m, 3H), 7.40 (d, *J* = 7.1 Hz, 1H), 7.27 (s, 1H), 7.02 (s, 1H), 6.47 (br s, 2H), 6.20 (t, *J* = 6.6 Hz, 1H), 6.00 (br s, 2H), 3.96 (q, *J* = 7.1 Hz, 2H), 3.80 (s, 3H), 3.74 (s, 3H), 3.61 (s, 2H), 2.59 (m, 2H), 1.09 (t, *J* = 7.1 Hz, 3H); ^13^C-NMR (100 MHz, DMSO-*d_6_*): δ 169.2, 165.7, 162.4, 161.1, 153.6, 152.5, 146.1, 142.5, 136.9, 136.2, 132.0 (2C), 128.8, 127.7, 126.4, 126.2, 123.5, 118.4, 117.7, 114.9, 106.2, 60.8, 60.3, 55.8, 47.8, 39.4, 32.3, 13.8. Anal. Calcd for C_28_H_30_N_6_O_5_·2.1 H_2_O: C, 59.17; H, 6.06; N, 14.79. Found: C, 59.16; H, 5.74; N, 14.60.

*Methyl (±)-(E)-2-(2-(3-(5-((2,4-Diaminopyrimidin-5-yl)methyl)-2,3-dimethoxyphenyl)acryloyl)-phthal-azin-2(1H)-yl)acetate* (**11c**). This compound was prepared as above using **5** (1.00 g, 2.59 mmol), **9c** (0.74 g, 2.85 mmol), *N*-ethylpiperidine (0.32 g, 0.40 mL, 2.85 mmol), and Pd(OAc)_2_ (20 mg, 0.089 mmol) in dry DMF (8 mL) to give **11c** (1.04 g, 78%) as a pale yellow solid, mp 158–160 °C. IR: 3477, 3370, 3192, 1720, 1653, 1614 cm^−1^; ^1^H-NMR (400 MHz, DMSO-*d*_6_): δ 7.98 (s, 1H), 7.87 (d, *J* = 15.9 Hz, 1H), 7.60 (d, *J* = 15.9 Hz, 1H), 7.59-7.46 (complex m, 4H), 7.39 (d, *J* = 7.1 Hz, 1H), 7.28 (s, 1H), 7.03 (s, 1H), 6.86 (br s, 2H), 6.37 (br s, 2H), 6.20 (t, *J* = 6.6 Hz, 1H), 3.80 (s, 3H), 3.75 (s, 3H), 3.62 (s, 2H), 3.51 (s, 3H), 2.60 (m, 2H); ^13^C-NMR (100 MHz, DMSO-*d*_6_): δ 169.6, 165.7, 162.8, 159.4, 152.5, 150.1, 146.2, 142.5, 136.9, 135.6, 132.01, 131.97, 128.8, 127.8, 126.3 (2C), 123.4, 118.6, 117.7, 115.0, 106.9, 60.8, 55.8, 51.6, 47.8, 32.1. Anal. Calcd for C_27_H_28_N_6_O_5_∙3.7 H_2_O: C, 55.61; H, 6.12; N, 14.41. Found: C, 55.63; H, 6.32; N, 14.43.

### 3.7. Biological Potency Measurements

Measurements of the MIC and the K_i_ utilized a racemic mixture of each compound and have been described previously [9,10,12,15,16]. In brief, MIC values were based on standardized cultures of *B. anthracis* Sterne strain as prescribed by the CLSI [19]. Evaluation of growth utilized spectrophotometric values of turbidity at 600 nm and on visual inspection for assessment of bacterial growth. The lowest concentration that yielded no growth was assigned as the MIC. Evaluation of the enzymatic activity and inhibition utilized purified DHFR protein cloned from *B. anthracis* Sterne strain and expressed recombinantly in *E. coli* BL21 (DE3) cells. The protein preparation utilized an *N*-terminal His-tag, which was determined to not interfere with the enzymatic activity assay and was left intact for the current studies. The reaction was reconstituted, including the NADPH co-factor, and was initiated by the addition of the dihydrofolate substrate. The reaction was carried out at 30 °C, and the linear rate was monitored for 2.8 min. These rates were plotted as a function of inhibitor concentration, and the 50% activity point was calculated using a 4-parameter curve fit. These IC_50_ values were converted to K_i_ values using the Cheng-Prusoff equation [20].

## 4. Conclusion

In summary, we have synthesized and evaluated a series of 11 new racemic dihydrophthalazine-bound 2,4-diaminopyrimidine-based compounds differing in substitution at C1 of the dihydrophthalazine moiety. From these new derivatives, compound **6e** bearing a cyclopropyl group at this position proved to be the most active compound generated to date, showing maximum potency against *B. anthracis* with respect to MIC and in K_i_ values compared to the other compounds prepared in this family. The strong correlation of these biological potency values suggests successful *in vivo* targeting of the DHFR enzyme. Derivatives possessing small alkyl groups, e.g. **6a** (R = Me) and **6b** (R = Et) also showed impressive potency. Finally, structures substituted with heteroaromatic rings (compounds **6f**–**h**) or acetic ester moieties (compounds **11a**–**c**) exhibited lower, though still significant, activity. The use of catalytic Bi(OTf)_3_ for both saponification and esterification reactions was successfully applied to the synthesis of the acetic esters explored in this study. Further investigations are underway to evaluate the biological activities of these drug scaffolds for other bacterial infections. 
